# Prognosis of patients with hepatocellular carcinoma treated with sorafenib: a comparison of five models in a large Canadian database

**DOI:** 10.1002/cam4.1493

**Published:** 2018-05-15

**Authors:** Haider H. Samawi, Hao‐Wen Sim, Kelvin K. Chan, Mohammad A. Alghamdi, Richard M. Lee‐Ying, Jennifer J. Knox, Parneet Gill, Adriana Romagnino, Eugene Batuyong, Yoo‐Joung Ko, Janine M. Davies, Howard J. Lim, Winson Y. Cheung, Vincent C. Tam, the CHORD Consortium

**Affiliations:** ^1^ British Columbia Cancer Agency Vancouver British Columbia Canada; ^2^ Princess Margaret Cancer Centre Toronto Ontario Canada; ^3^ Sunnybrook Odette Cancer Centre Toronto Ontario Canada; ^4^ King Saud University Riyadh Saudi Arabia; ^5^ Tom Baker Cancer Centre Calgary Alberta Canada

**Keywords:** ALBI, BCLC, CLIP, HCC, Okuda, prognostic models, TNM

## Abstract

Several systems (tumor‐node‐metastasis [TNM], Barcelona Clinic Liver Cancer [BCLC], Okuda, Cancer of the Liver Italian Program [CLIP], and albumin–bilirubin grade [ALBI]) were developed to estimate the prognosis of patients with hepatocellular carcinoma (HCC) mostly prior to the prevalent use of sorafenib. We aimed to compare the prognostic and discriminatory power of these models in predicting survival for HCC patients treated with sorafenib and to identify independent prognostic factors for survival in this population. Patients who received sorafenib for the treatment of HCC between 1 January 2008 and 30 June 2015 in the provinces of British Columbia and Alberta, and two large cancer centers in Toronto, Ontario, were included. Survival was assessed using the Kaplan–Meier method. Multivariate Cox regression was used to identify predictors of survival. The models were compared with respect to homogeneity, discriminatory ability, monotonicity of gradients, time‐dependent area under the curve, and Akaike information criterion. A total of 681 patients were included. 80% were males, 86% had Child–Pugh class A, and 37% of patients were East Asians. The most common etiology for liver disease was hepatitis B (34%) and C (31%). In all model comparisons, CLIP performed better while BCLC and TNM7 performed less favorably but the differences were small. The utility of each system in allocating patients into different prognostic groups varied, for example, TNM poorly differentiated patients in advanced stages (8.7 months (m) (95% CI 6.5–11.5) versus 8.4 m (95% CI 7.0–9.6) for stages III and IV, respectively) while ALBI had excellent discrimination of early grades (15.6 m [95% CI 13.0–18.4] versus 8.3 m [95% CI 7.0–9.2] for grades 1 and 2, respectively). On multivariate analysis, hepatitis C, alcoholism, and prior hepatic resection were independently prognostic of better survival (*P *<* *0.01). In conclusion, none of the prognostic systems was optimal in predicting survival in sorafenib‐treated patients with HCC. Etiology of liver disease should be considered in future models and clinical trial designs.

## Introduction

Hepatocellular carcinoma (HCC) is the most common primary malignancy of the liver and the second leading cause of cancer‐related mortality worldwide 1. Despite this high global burden, limited treatment options exist for advanced disease. In recent years, sorafenib, an oral multityrosine kinase inhibitor that inhibits growth of the tumor and its vasculature, has been shown to improve overall survival (OS) by 2–3 months in two large randomized phase III clinical trials 2, 3 and is considered the standard treatment for patients with advanced HCC who have adequate liver function and who are otherwise ineligible for local therapies. Nonetheless, its modest survival benefit must be weighed against the potential for significant toxicities. The ability to better identify patients who would benefit from sorafenib therapy remains a challenge because predictive biomarkers for efficacy of sorafenib are scarce. The magnitude of benefit also varies and appears to be influenced by the etiology of the underlying liver disease 4, 5. Consequently, best supportive care remains a reasonable alternative for a select group of patients with poor prognosis. A prognostic scoring system would help guide physicians to identify those patients in which the potential value of intensive therapy outweighs its burden 6. Additionally, identifying relevant prognostic factors is essential in stratifying patients for future clinical trials.

The prognosis of patients with HCC is complex and uniquely influenced by the severity of hepatic dysfunction. Prior studies have attempted to improve on the prognostic power of the traditional tumor‐node‐metastasis (TNM) staging system by identifying and incorporating other relevant factors 7, 8. However, ongoing debate exists as to which staging system is the most informative and whether any of these systems are suitable for HCC patients treated with sorafenib. The most commonly used staging systems are TNM (7th edition at the time of this study, TNM7) 9, Barcelona Clinic Liver Cancer (BCLC) 10, 11, Cancer of the Liver Italian Program (CLIP) 7, 12, and Okuda 13 (Table 1). A fifth system, the ALBI grade 14, is a novel system that objectively assesses liver function using albumin and bilirubin for all stages of HCC and represents a simple alternative to the Child–Pugh (CP) classification. With the exception of ALBI grade, all the other systems were developed prior to the prevalent use of sorafenib in the treatment of advanced HCC and have not been evaluated specifically in this population.

**Table 1:** Components of HCC staging and prognostic systems

Systems	PS	Tumor‐related factors				Liver function			AFP	Prior Tx	LiverDx^b^
		Size	Nodes	PVT	Extent^a^	CP	Alb	Bili			
TNM7	✖	✔	✔	✔	✖	✖	✖	✖	✖	✖	✖
BCLC	✔	✔	✖	✔	✖	✔	✖	✔	✖	✖	✖
CLIP	✖	✔	✖	✔	✔	✔	✖	✔	✔	✖	✖
Okuda	✖	✔	✖	✖	✔	✖	✔	✔	✖	✖	✖
ALBI	✖	✖	✖	✖	✖	✖	✔	✔	✖	✖	✖

AFP, alpha‐fetoprotein; Alb, albumin; ALBI, albumin–bilirubin grade; BCLC, Barcelona Clinic Liver Cancer; Bili, bilirubin; CLIP, Cancer of Liver Italian Program; CP, Child–Pugh classification; HCC, hepatocellular carcinoma; PS, performance status; PVT, portal vein thrombosis; TNM7, TNM staging seventh edition; Tx, treatment.

a Extent of primary tumor compared to liver area (greater or less than 50%).

b Etiology of liver disease.

The objectives of this study were to compare the utility of five commonly used staging systems to predict survival and to identify independent prognostic factors for survival in a large multicenter cohort of HCC patients treated with sorafenib. Our primary hypothesis was that none of the currently used staging systems is ideal in stratifying sorafenib‐treated patients with HCC and that identification of new prognostic factors that are unique to this population is needed.

## Patients and Methods

### CHORD consortium

The Cancer Health Outcomes Research Database (CHORD) consortium is a national initiative in Canada that brings together a group of cancer researchers with the aim of pooling and merging diagnostic, treatment, and prognostic data into a large database for research. This pooled data source is particularly useful to study tumors that are rare and difficult to treat, and where sample size from any single institution may not be adequate to address a clinical research query. Common predefined data elements are collected from each participating center and then merged and standardized into a central repository prior to analysis. For this study, data were limited to three large provinces due to data availability and study timeframes. Ethics approval was obtained from each participating center prior to the conduct of this study.

### Study setting

Patients included in this study were treated at cancer centers from three Canadian provinces; British Columbia, Alberta, and Ontario. The study involved all the cancer centers operated by the BC Cancer Agency (BCCA) and Alberta health Services (AHS), and two large comprehensive cancer centers (Princess Margaret Cancer Centre [PMCC] and Sunnybrook Odette Cancer Centre [OCC]) in Toronto, Ontario.

### Data collection

The pharmacy databases from the BCCA, AHS, PMCC, and OCC were queried for all patients who received at least one dose of sorafenib for the treatment of advanced HCC during the time period between 1 January 2008 and 30 June 2015. Diagnosis of HCC was made based on histologic confirmation or fulfillment of radiologic criteria according to the American Association for Study of Liver Disease (AASLD) 15, 16. For each patient, the electronic medical record (EMR) was reviewed in order to collect patient demographics, clinical data, and treatment characteristics.

Staging was determined based on the most recent radiologic imaging prior to receipt of first sorafenib dose. Intrahepatic disease was evaluated using triphasic computed tomography (CT) or magnetic resonance imaging (MRI) of the liver. Tumor characteristics as described by the radiology report were collected and analyzed. The CP classification was used for assessment of hepatic function.

For each patient, all prior treatment modalities were recorded. These treatments included surgery, tumor embolization (trans‐arterial chemombolization [TACE], trans‐arterial radioembolization [TARE], and bland embolization), tumor ablation (radiofrequency ablation [RFA] and alcohol injection), or using stereotactic body radiation (SBRT). In addition, information on sorafenib starting dose, dosing adjustment, and toxicities was collected and will be reported separately. Patients were excluded if they did not receive sorafenib or did not have adequate follow‐up information.

### Statistical analysis

The baseline characteristics of the study population were summarized using descriptive statistics. In order to identify independent prognostic factors, univariate analyses were performed on all baseline characteristics. Subsequently, factors that were significant on univariate analyses (*P *<* *0.1) were used to construct a multivariate Cox regression model using a stepwise forward selection approach. In this model, patients with less common or multiple etiologies for liver diseases were classified into one group. OS was defined as the time interval from the start of sorafenib therapy to the date of death from any cause, with censoring on the date of last follow‐up.

Individual scores based on all five staging systems (TNM7, BCLC, CLIP, Okuda, and ALBI) were retrospectively derived for each patient based on the pooled clinical, radiological, and biochemical data in CHORD.

Three criteria are commonly used to assess the performance and utility of staging systems: (1) Homogeneity: Patients in the same stage have similar survival, (2) Discriminatory ability: Patients in different stages within the system have greater differences in their survival, and (3) Monotonicity of gradients: Patients in earlier stages will always have better survival compared with those with more advanced stages 17, 18. To measure homogeneity, we used the likelihood ratio chi‐square (LR *χ* ^2^) in Cox regression 19. Linear trend chi‐square^,^ assuming ordinal groups in Cox regression, was used to measure the discriminatory power of each staging system 20. Both the LR chi‐square and linear trend chi‐square tests 17, 18 were also used to assess the monotonicity of the gradients of survival with 1 degree of freedom. A higher test statistic with a statistically significant *P*‐value means the prognostic system is better. In addition, model fit statistics using the Akaike information criterion (AIC) was used to measure the discriminatory ability because it accounts for model complexity and compares between models irrespective of the absolute prognostic power of each individual model 21, 22. A lower AIC means that the model is more informative. Cox proportional hazards assumption was assessed through graphical approach examining the log–log plots. To further assess the predictive and discriminatory power of these prognostic scores, we also performed time‐dependent receiver operator characteristics (ROC) curve survival analysis via the R “timeROC” package, which uses the inverse probability censoring weighting (IPCW) method without competing risks 23. A few time points were constructed to produce the time‐specific area under the curve (t‐AUC) summaries where a higher t‐AUC represents better predictive power.

For all analyses, a two‐tailed *P *<* *0.05 was considered statistically significant. Statistical analysis was performed using SAS v9.4 (SAS, Inc., Cary, NC), R version 3.3.0, and SPSS version 24.0.

## Results

### Description of study population

A total of 681 patients with HCC who received treatment with sorafenib were identified and included, of which 643 had complete data on all five prognostic systems. The largest number of patients was from the BCCA (288 [42%]), followed by PMCC (215 [32%]), while AHS and OCC contributed 155 (23%) and 23 (3%) patients, respectively. Table 2 summarizes the baseline characteristics of the patients included in this study. In the entire cohort, median age from initiation of sorafenib was 64 (IQR 58–73) years. Pathologic confirmation of HCC was obtained in 60% of patients, 80% were males, and 37% were East Asian. Preexisting liver cirrhosis was present in 70% of the patients, and most patients (86%) had CP class A at the time of sorafenib initiation. The most common etiology of liver disease was hepatitis B (HBV), hepatitis C (HCV), and alcoholism (34%, 31%, and 23%; respectively). Furthermore, 102 (15%) patients had multiple preexisting etiologies of whom 66 patients had history of alcohol consumption combined with another liver disease. With respect to other treatments, the majority of the patients (59%) received at least one mode of locoregional therapy prior to sorafenib commencement, most commonly TACE, liver resection, and ablation (33%, 24%, and 22%, respectively). In contrast, only a minority of patients (22%) received antineoplastic treatment following sorafenib cessation irrespective of the reason for discontinuation.

**Table 2:** Baseline characteristics of HCC patients treated with sorafenib

Characteristics	*N* = 681 (%)
Age, median (IQR)	64 (58–73)
<60	219 (32)
61–70	239 (35)
>70	223 (33)
Gender	
Male	547 (80)
Female	134 (20)
Ethnicity	
East Asian	250 (37)
Other	431 (63)
Liver disease etiology	
Hepatitis B	229 (34)
Hepatitis C	209 (31)
Alcohol‐related	159 (23)
Other	74 (11)
None	112 (16)
Liver cirrhosis	480 (70)
ECOG PS	
0/1	595 (87)
2/3	82 (12)
Unknown	4 (1)
Child–Pugh	
A	585 (86)
B	92 (13)
C	1 (<1)
T stage	
T1/2	225 (33)
T3/4	380 (56)
Unknown	76 (11)
*N* stage	
N0	449 (66)
N1	205 (30)
Unknown	27 (4)
M stage	
M0	352 (52)
M1	320 (47)
Unknown	9 (1)
PVT	
Yes	272 (40)
No	403 (59)
Unknown	6 (1)
Tumor extension	
>50%	206 (30)
<50%	464 (68)
Unknown	11 (2)
Number of tumors	
0	40 (6)
1–4	313 (46)
Multifocal	309 (45)
Unknown	19 (3)
Prior treatments number	
0	278 (41)
1	221 (32)
2+	182 (27)
Type	
Liver resection	163 (24)
Ablation	148 (22)
TACE	227 (33)
TARE	22 (3)
SBRT	37 (5)
Alcohol injection	24 (4)
Transplant	33 (5)
Subsequent treatment	
Yes	144 (22)
No	520 (76)
Unknown	17 (2)

ECOG, Eastern Cooperative Group performance status; HCC, hepatocellular carcinoma; IQR, interquartile range; PVT, portal vein thrombosis; SBRT, stereotactic body radiation; TACE, trans‐arterial chemoembolization; TARE, trans‐arterial radioembolization.

### Survival across the prognostic systems

**Figure 1:**
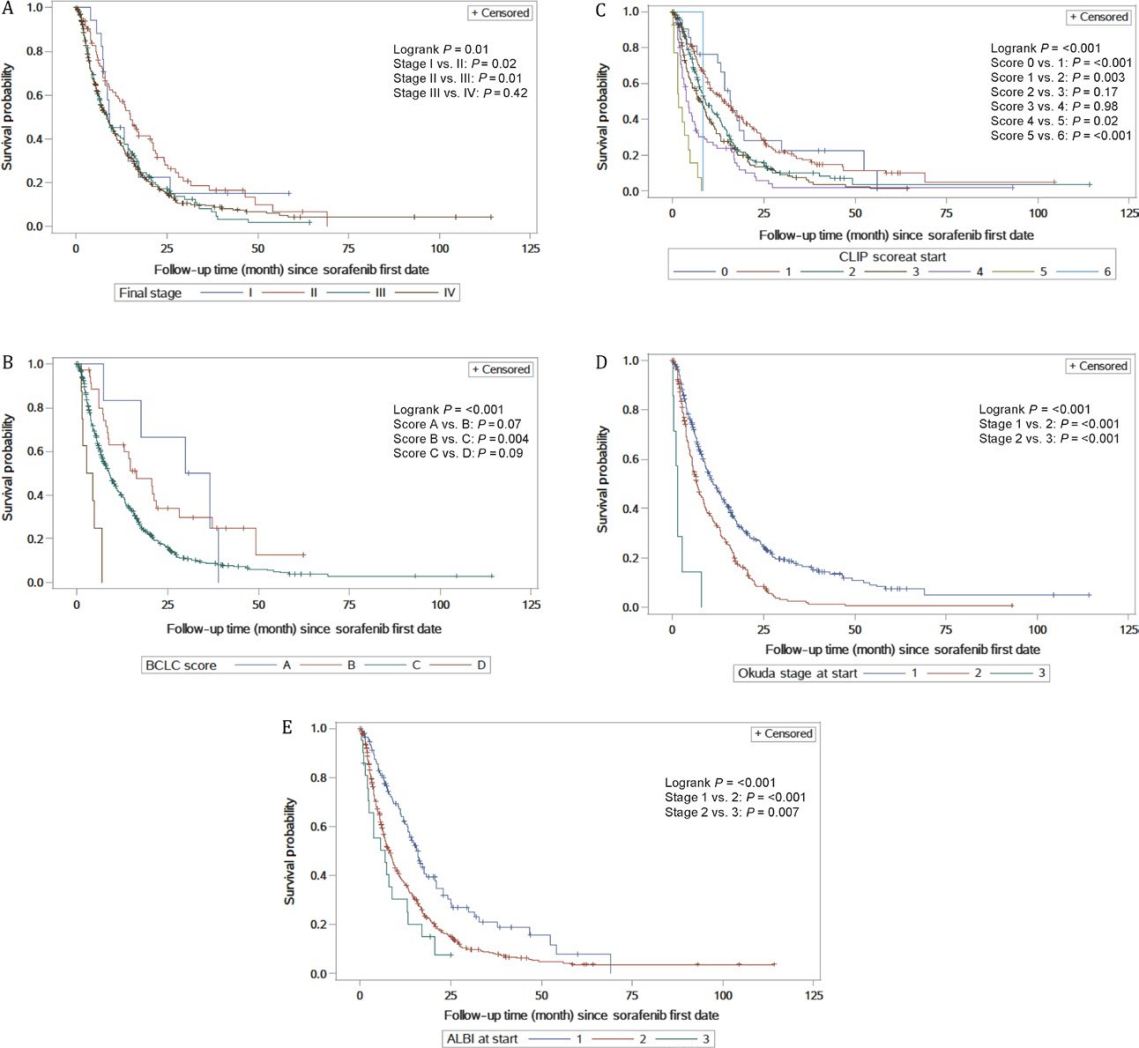
Kaplan–Meier survival curves for each staging system: A (TNM7), B (BCLC), C (CLIP), D (OKUDA), and E (ALBI). TNM7, TNM staging seventh edition; BCLC, Barcelona Clinic Liver Cancer; CLIP, Cancer of Liver Italian Program; ALBI, albumin–bilirubin grade.

At the time of this analysis, 539 (79%) patients had died. The median follow‐up was 37.6 (IQR 29.5–41.1) months, and the median OS (mOS) for the entire cohort was 9.2 (95% CI 8–10.4) months. The five systems were analyzed using the Kaplan–Meier (KM) method, and the results are illustrated in Figure 1. In general, all systems were able to stratify patients into different prognostic groups (overall *P* value <0.01). Nonetheless, the performance within each model was not universally consistent. For instance, TNM was limited in stratifying patients in the more advanced stages (stages III and IV). Likewise, CLIP showed overlap in survival curves for the intermediate stages (scores 2–4). In contrast, the BCLC, Okuda, and ALBI systems showed better performance in prognostication across all stages with a significant *P* value for all stage‐to‐stage comparisons (*P *<* *0.05). Further details on the distribution of patients and mOS by stage within each system are summarized in Table 3.

**Table 3:** Patient distribution and median overall survival according to stage at start of sorafenib treatment

Systems	*N* = 643 (%)	mOS months (95% CI)
TNM7 staging		
I	18 (3)	9.4 (7.2–17.3)
II	83 (13)	15.1 (10.1–20.9)
III	148 (23)	8.7 (6.5–11.5)
IV	394 (61)	8.4 (7.0–9.6)
BCLC		
A	7 (1)	33.2 (7.2–38.9)
B	37 (6)	16.4 (8.6–28.3)
C	591 (92)	8.9 (7.9–10.3)
D	8 (1)	3.7 (1.2–6.9)
CLIP		
0	22 (3)	16. 0 (12.3–19.6)
1	163 (25)	13.7 (10.3–17.3)
2	215 (33)	9.1 (7.5–12.1)
3	162 (25)	8.0 (5.5–9.3)
4	66 (10)	4.5 (2.9–6.2)
5	14 (2)	1.8 (0.5–4.5)
6	1 (<1)	8.6
Okuda		
1	364 (57)	11.2 (9.6–13.5)
2	272 (42)	6.8 (5.7–8.4)
3	7 (1)	1.5 (0.2–2.9)
ALBI		
1	119 (19)	15.6 (13.0–18.4)
2	503 (78)	8.3 (7.0–9.2)
3	21 (3)	6.9 (2.3–12.9)

ALBI, albumin–bilirubin grade; BCLC, Barcelona Clinic Liver Cancer; CLIP, Cancer of Liver Italian Program; HCC, hepatocellular carcinoma; mOS, median overall survival; TNM7, TNM staging seventh edition.

### Comparison of the performance of prognostic systems

All the comparisons between the prognostic systems are summarized in Table 4. CLIP followed by Okuda had the highest homogeneity and the highest discriminatory ability with a significant *P* value. Monotonicity of gradients was maintained within all staging systems, with the exception of TNM7 (Fig. 1). Using both t‐AUC and AIC, CLIP and Okuda were ranked highest (t‐AUC = 0.659, 95% CI 0.601–0.718 and 0.645, 95% 0.597–0.694, respectively) and (AIC = 5725.76 and 5730.38, respectively). TNM7, BCLC, and ALBI ranked lower according to both models. Nonetheless, there was no significant difference between all five models when assessed using t‐AUC.

**Table 4:** Comparison of staging systems for HCC patients treated with sorafenib using t‐AUC at 24 months, AIC, homogeneity (LR *χ* ^2^), and linear trend chi‐square tests

Staging systems	Homogeneity (LR χ^2^) (*P* value)	Linear trend χ^2^ (*P* value)	t‐AUC (95% CI)	AIC
CLIP	63.37 (<0.001)	54.26 (<0.001)	0.66 (0.60–0.72)	5725.76
Okuda	50.76 (<0.001)	44.27 (<0.001)	0.64 (0.60–0.69)	5730.38
ALBI	24.40 (<0.001)	23.93 (<0.001)	0.56 (0.51–0.60)	5756.73
BCLC	23.88 (<0.001)	20.09 (<0.001)	0.56 (0.52–0.60)	5759.25
TNM7	11.63 (0.009)	8.44 (0.004)	0.56 (0.50–0.62)	5771.51

AIC, Akaike information criterion; ALBI, albumin–bilirubin grade; BCLC, Barcelona Clinic Liver Cancer; CLIP, Cancer of Liver Italian Program; HCC, hepatocellular carcinoma; t‐AUC, time‐dependent area under the curve; TNM7, TNM staging seventh edition.

### Predictors of survival

Baseline factors that were included in univariate analysis were as follows: age, gender, ethnicity, performance status (PS) as measured by the Eastern Cooperative Oncology Group (ECOG), alpha‐fetoprotein (AFP), etiology of liver disease, stage, extent of primary tumor, portal vein thrombosis (PVT), CP classification, and prior treatment modalities. On multivariate analysis (Table 5), poor PS (ECOG 2–3) and more extensive liver disease (CP class B or involvement of >50% of liver) correlated with a significantly higher risk of death (HR 2.01, 95% CI 1.60–2.77; HR 1.80, 95% CI 1.37–2.36 and HR 1.32, 95% CI 1.07–1.61, respectively). Conversely, PVT and stage were not significantly associated with survival. Patients with preexisting HCV and alcohol‐related liver disease had a lower risk of death when compared to no history of liver disease (HR 0.61, 95% CI 0.46–0.83 and HR 0.61 95% CI 0.42–0.87, respectively). Likewise, prior hepatic resection correlated favorably with survival (HR 0.54, 95% CI 0.43–0.69).

**Table 5:** Independent prognostic factors for overall survival in HCC patients treated with sorafenib according to multivariate analysis

Variables	HR	95% CI	*P* value
ECOG PS 2–3 versus 0–1	2.01	1.60–2.77	<0.001
Child Pugh B versus A	1.80	1.37–2.36	<0.001
Etiology of liver disease			
None (Reference)			
Hepatitis B	0.87	0.64–1.13	0.26
Hepatitis C	0.61	0.46–0.83	0.001
Alcohol‐related liver disease	0.61	0.42–0.87	0.006
Other/multiple	0.63	0.45–0.88	0.007
Extent of tumor within the liver			
>50% versus <50%	1.32	1.07–1.61	0.008
AFP > ULN	1.47	1.20–1.80	<0.001
Prior hepatic resection	0.54	0.43–0.69	<0.001

AFP, alpha‐fetoprotein; ECOG, Eastern Cooperative Group performance status; HCC, hepatocellular carcinoma; ULN, upper limit of normal.

## Discussion

In this large multicenter study, we examined the role of several commonly used staging systems in classifying patients with HCC treated with sorafenib in an attempt to determine the most informative staging system. When compared to each other, CLIP showed superior performance in predicting survival. However, the differences between the staging systems were modest, and none of them emerged as the optimal choice. Finally, additional prognostic factors, not included in former models, were identified on multivariate analysis and should be considered when developing future models.

CLIP score is a commonly used system that incorporates liver function and tumor characteristics. It has been externally validated in both East Asian and Western populations 18, 22, 24. When compared to the other models, CLIP was the best system in terms of homogeneity within the same stage, monotonicity of gradients and had the highest predictive ability. This finding is supported by previous studies 25, 26 but challenged in another report 27. However, the latter study was limited by a small number of patients, and it was restricted to East Asian patients with HCC. Nonetheless, CLIP was inadequate in prognosticating patients in the intermediate stages. One explanation might be that it lacks assessment of PS. Correlation of PS with survival in HCC has been shown in other studies 8, 28, and a good PS has been a prerequisite for inclusion in major clinical trials that involved the use of sorafenib 2, 3. Additionally, improving the prognostic ability of CLIP by the addition of PS has been suggested 26.

In this study, we demonstrate that TNM was insufficient in predicting survival for patients with HCC treated with sorafenib, and the majority of patients in this cohort had advanced disease (stages III and IV). In addition, KM curves showed lack of correlation between stage and survival. Our findings are consistent with published reports on the limited utility of TNM in stratifying patients with advanced HCC 25.

BCLC is the most commonly used system in Western countries and endorsed by the European Association for the Study of the Liver (EASL) 29 and AASLD 30. BCLC provided the best prognostic stratification in multiple studies, particularly in patients with cirrhosis and after radical therapies 17. In contrast, BCLC was not as useful in our cohort possibly because the vast majority of patients who received sorafenib had BCLC stage C which includes a heterogeneous group of patients with CP class A or B, presence of PVT or metastatic disease irrespective of the number, nature or extent of the hepatic tumors 29. Previous studies have also shown the limited prognostic utility of BCLC in advanced HCC 25, 26, and attempts to further stratify stage C have been published 31, 32.

Hepatic dysfunction has a critical impact on survival in HCC irrespective of stage. Unlike TNM staging, which depends purely on anatomic extent of the tumor, the ALBI grade belongs on the other end of the spectrum and uses only albumin and bilirubin as a measure of hepatic function. The use of laboratory parameters reduces subjectivity, which is often a criticism of the CP classification. The ALBI grade, which allocates patients into one of three prognostic categories, was initially developed in Japanese patients with HCC across all stages. It was subsequently validated in multiple geographic locations and in different clinical settings, including sorafenib‐treated patients and those undergoing resections 14. In our cohort, there was a nearly 7‐month difference in mOS between ALBI grades 1 and 2. Our real‐world data show similar findings to the original report by Johnson et al. in which clinical trial patients with advanced HCC and CP class A who received sorafenib had an almost 6‐month difference in mOS when classified into “good risk group (ALBI grade 1)” and “poorer risk group (ALBI grade 2)” 14. The use of these groups to guide treatment decisions and to stratify patients in clinical trials needs further evaluation. Nonetheless, these data suggest that hepatic function alone has the most significant impact on survival in advanced HCC.

Patients with advanced HCC have poor prognosis, and most patients will die within one year of diagnosis. In this large multicenter analysis, the mOS was 9.2 months, but there are substantial differences in survival estimates among different studies. In the two pivotal trials examining the benefit of sorafenib in patients with, mOS in the SHARP trial (10.7 months) 3 was superior to that in the Asia–Pacific trial (6.5 months) 2 despite similar inclusion and exclusion criteria. Similarly, survival varied in other reports 4, 25, 33, 34 which are likely due to the heterogeneous nature of this group of patients and the differences in patient and disease characteristics across different geographic locations and practice settings.

Several independent prognostic variables were identified in this analysis. As expected, factors such as PS and liver function impairment were strongly associated with the risk of death. A system that incorporates assessment of the extent of the tumor, the degree of hepatic dysfunction, and the overall condition of the patient could potentially overcome some of the limitations of existing systems. Interestingly, while the degree of hepatic dysfunction plays a major role in most prognostic systems, only BCLC includes assessment of PS. In fact, the addition of PS to CLIP might be associated with improvement in its discriminatory ability, making it a good candidate for designing a new model 25. Other important factors that appeared to have a favorable impact on survival are HCV, alcohol‐related liver disease, and prior surgical resection or ablation. The natural history and response to therapy in HCC appear to be influenced by the underlying cause of liver disease. HCV‐related HCC has been correlated with better outcomes compared with other subgroups and particularly when compared to HCC caused by HBV 4, 5, 35. HCV core proteins were shown to result in constitutive activation of Raf‐1 kinase 36, and therefore, it has been hypothesized that the antineoplastic effects of sorafenib, which blocks the activity of Raf‐1 kinase, are more pronounced in HCV‐related HCC. However, despite lower OS in HBV‐related HCC, these patients seem to continue to derive benefit from sorafenib therapy 37. Our study lends further evidence in support of stratifying patients according to etiology of liver disease in prospective clinical trials of advanced HCC.

Our study has several strengths as well as limitations. The main strength is its large multicenter design which included significant numbers of both Western and East Asian patients with a variety of liver disease etiologies all of whom were treated similarly at different Canadian cancer centers. This addressed common limitations in a number of published retrospective studies 17, 22, 25, 27. A further advantage is that the staging systems were evaluated with respect to their performance and overall predictive value. Nonetheless, readers should interpret the findings in the context of several limitations. First, this is a retrospective nonrandomized study and thus prone to selection bias. For instance, the majority of patients had good PS (88% ECOG 0/1), which could lead to overestimation of survival. However, this reflects routine practice where sorafenib is more commonly offered to patients with good general function. Second, our sample is limited to patients treated at cancer centers and therefore excluded patients not referred for medical or logistical reasons. Third, we did not include liver enzyme elevations such as increased aminotransferases (AST and ALT) because these laboratory values are not consistently captured in our database. However, aminotransferase levels are not helpful in determining underlying liver disease, and an elevated AST/ALT ratio is more indicative of the presence of liver cirrhosis 38. Finally, continuous improvements in supportive care measures and cumulative experience with sorafenib use and toxicity could have affected the duration of treatment and survival; however, it is challenging to control for such supportive care changes over time in retrospectively designed studies.

## Conclusions

In HCC patients treated with sorafenib, a system that considers all known major prognostic factors is lacking. Among five commonly used staging systems, CLIP was the most useful in predicting survival while BCLC and TNM7 had limited benefit in this population. This analysis, although not designed to be a validation study, provided real‐world evidence to support the use of ALBI grade to stratify patients into prognostic risk groups that could be used to guide patient counseling and treatment decisions. Further, our study showed that the etiology of liver disease has a considerable impact on the trajectory of HCC and possibly on its response to therapy. Therefore, these factors should be considered in future prognostic models as well as in the design and stratification of patients in future randomized clinical trials.

## Conflict of Interest

There is no conflict of interest for all the authors.
